# Patterns of Ant (Hymenoptera: Formicidae) Richness and Relative Abundance along an Aridity Gradient in Western Venezuela

**DOI:** 10.1007/s13744-012-0096-y

**Published:** 2012-12-21

**Authors:** A J Pérez-Sánchez, J E Lattke, A L Viloria

**Affiliations:** 1 Lab de Biología de Organismos, Centro de Ecología, Instituto Venezolano de Investigaciones Científicas (IVIC), Caracas, Venezuela; 2 Museo del Instituto de Zoología Agrícola, Univ Central de Venezuela, Maracay, Venezuela; 3 Lab de Ecología Sensorial, Centro Multidisciplinario de Ciencias, Instituto Venezolano de Investigaciones Científicas (IVIC—Mérida), Apartado 20632, Merida, 1020-A Venezuela

**Keywords:** Ant diversity, Araya Peninsula, Mantel correlograms, neotropics, semi-arid environments

## Abstract

In xeric ecosystems, ant diversity response to aridity varies with rainfall magnitude and gradient extension. At a local scale and with low precipitation regimes, increased aridity leads to a reduction of species richness and an increased relative abundance for some ant species. In order to test this pattern in tropical environments, ant richness and relative abundance variation were evaluated along 35 km of an aridity gradient in the Araya Peninsula, state of Sucre, Venezuela. Three sampling stations comprising five transects each were set up. Pitfall traps and direct collecting from vegetation were assessed per transect. Overall, 52 species, 23 genera, and 7 subfamilies of ants were recorded in the peninsula. The total number of species and genera recorded by both sampling stations and transects decreased linearly with increasing aridity. Total relative abundance was highest in the most arid portion of the peninsula, with *Crematogaster rochai* (Forel) and *Camponotus conspicuus zonatus* (Emery) (Hymenoptera: Formicidae) being the numerically dominant species. Spatial and multivariate analyses revealed significant changes in ant composition every 11 km of distance, and showed a decrease of ant diversity with the increase of harsh conditions in the gradient. Here, we discuss how local geographic and topographic features of Araya originate the aridity gradient and so affect the microhabitat conditions for the ant fauna.

**Electronic supplementary material:**

The online version of this article (doi:10.1007/s13744-012-0096-y) contains supplementary material, which is available to authorized users.

## Introduction

Changes in ant diversity along broad-scale transitions, ecotones, and gradients have been an important objective of myrmecological studies in arid environments during the last decades (Andersen 1997, Pfeiffer *et al* 2003, Reznikova 2003, Dunn *et al* 2009). Ant variation along aridity gradients have been assessed through several approaches, ranging from natural transition evaluations to habitat disturbance studies, including altitudinal and precipitation gradient surveys (Andersen 1997, Kaspari *et al* 2000, Bestelmeyer & Wiens 2001, Araújo & Fernandes 2003, Pfeiffer *et al* 2003, Sanders *et al* 2003, Gunawardene & Majer 2004, Delsinne *et al* 2010). In general, aridity gradients are correlated with significant changes in vegetative component and coverage as a consequence of the precipitation and temperature variation effect on net primary productivity (Schulze *et al* 1996, Scholes *et al* 2002, Tieleman *et al* 2003, Petrů *et al* 2006). Although animal richness tends to increase along with such variables within aridity gradients (Mittelbach *et al* 2001), the response of ant assemblages to aridity variations depend upon rainfall intensity and scale of the study (Davidson 1977, Morton & Davidson 1988, Medel 1995, Kaspari *et al* 2000, Delsinne *et al* 2010). However, several studies have revealed that rising aridity leads to a reduction in ant richness, as well as an increase in the abundance of some species within ant assemblages (Whitford 1978, Marsh 1986, Sanders *et al* 2003, Gunawardene & Majer 2004).

**Fig 1:**
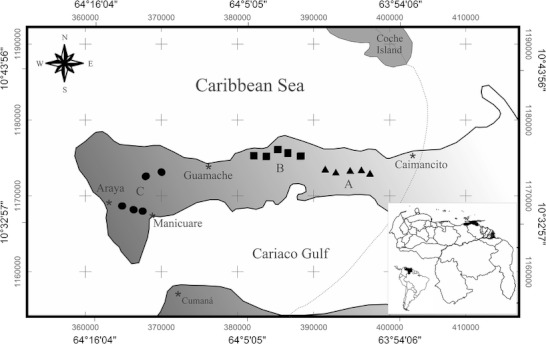
Geographic location of the Araya Peninsula, State of Sucre, Venezuela. The map shows the location of sampling stations (*A*, *B*, and *C*) and transects (*geometric symbols*) within the aridity gradient (*gray area*). The *dotted line* indicates the precipitation change suggested by Thielen (2003). The *gray* values are UTM geographic coordinates (zone 20).

The Araya Peninsula is one of the driest localities in Venezuela, with some years of no precipitation at all (Cumana 1999, Thielen 2003). Although there are no accurate rainfall records along the peninsula’s extension, several authors recognize an aridity gradient from east to west based on the erratic behavior of precipitation, temperature variation, and significant changes in the vegetation (Guevara *et al* 1992, Cumana 1999, González *et al* 2004). According to climatic estimations by Thielen (2003), this decrease in rainfall starts from near of the peninsula base (Caimancito town) towards the western tip of the region (Fig 1). In the last 20 years, there have been several publications of faunistic and floristic information for this region, mainly the eastern side (Guevara *et al* 1992, Lentino & Bruni 1994, Cumana 1999, Cumana *et al* 2000, Cornejo & Prieto 2001, Cumana & Cabeza 2003, Leopardi *et al* 2009). However, information about biotic turnover along the peninsula is limited to lizard fauna (González *et al* 2004), while functionally important groups, such as ants, are completely unknown. Therefore, the aim of this work was to evaluate ant richness and relative abundance changes along the aridity gradient of the Araya Peninsula, State of Sucre, Venezuela. Considering the premise that aridity limits ant diversity in terms of resource availability, physiological stress, and increased competition (Andersen 1997, Bestelmeyer 2000, Pfeiffer *et al* 2003, Reznikova 2003, Sanders *et al* 2003), we expect a decrease of ant richness and a higher presence of numerically dominant species (sensu Davidson 1998) in proportion to the increasing aridity gradient from east to west. To test this hypothesis, we evaluated changes in these parameters with a taxonomic (species and genera) and spatial (sampling stations and transects) approach along 35 km of gradient (Fig 1).

## Material and Methods

The Araya Peninsula is located in State of Sucre, eastern Venezuela (Fig 1). It occupies 652 km^2^ with a length of 60 km and a width between 4 and 24 km (Cumana 1999, González *et al* 2004). There are a series of low and narrow central hills that increase in altitude from west (<100 m) to east, reaching their highest point (600 m) in the southeastern peninsula (Ewel & Madriz 1976, Cumana 1999, González *et al* 2004). The climate is arid at the western edge and semi-arid toward the east due to the combined effect of maritime-continental winds and topography (Ewel & Madriz 1976, Cumana 1999, González *et al* 2004). Given the absence of meteorological stations in the peninsula, the annual mean precipitation is either frequently cited, calculated, or extrapolated from the 1984–1990 records of the Guayacán, Cumaná, Cariaco, and San Pedro de Coche weather stations (Guevara *et al* 1992, Leopardi *et al* 2009). The annual mean precipitation varies between 500 and 800 mm at the peninsula base, and decreases to 243.8 mm towards the western tip (Guevara *et al* 1992, Thielen 2003, Leopardi *et al* 2009). There are eight dry months during August–November and February–May, and two periods of precipitation during December–January and June–July (Guevara *et al* 1992). The annual mean temperature is 27.3°C and soil textures vary from loamy clays to loamy silt (Matteucci 1986, Leopardi *et al* 2009).

The vegetation is represented by 221 recorded angiosperm species that form four general landscape types: coastal herb plains, mangroves, savanna, and xerophilous shrublands (Cumana 1999, González *et al* 2004). Coastal herb plains have halophytic and psammophylic species which represent the lower strata along the northern coast (Cumana 1999). Mangroves correspond to woody formations located in wet and salty soils, which form small, isolated, and dispersed groups of *Rhizophora mangle* (Rhizophoraceae), *Avicennia germinans* (Acanthaceae), *Conocarpus erectus*, and *Laguncularia racemosa* (Combretaceae) (Cumana 1999). The Savanna is located in the central region of the peninsula and is dominated by the grasses *Aristida*, *Bouteloua*, *Digitaria*, *Eragrostis*, *Paspalum*, and *Trachypogon* (Cumana 1999). This vegetative formation exhibits a poor but well-defined arboreal stratum: *Byrsonima crassifolia* (Malpighiaceae), *Mabea occidentalis* (Euphorbiaceae), and *Roupala montana* (Proteaceae).

The xerophilous shrubland is the dominant formation and is mostly woody plants, trees, shrubs, vines, and epiphytic–hemiparasitic species, with a clear predominance of cacti and deciduous armed legumes in the canopy (Cumana 1999). Height and abundance of woody species allow recognizing two physiognomies, cactus scrubs and thorny shrublands (González *et al* 2004). The former is located from the western tip to Chacopata hill, and is characterized by legume species with heights below 5 m and columnar cacti that barely reach 3 m (González *et al* 2004). The thorny shrubland distributed from the central region to the eastern side of the peninsula, and has a greater proportion of woody and succulent cacti that surpass 8 m in height (González *et al* 2004). Both vegetation types have abundant *Prosopis juliflora* (Mimosaceae) and *Caesalpinia granadillo* (Caesalpinaceae), as well as *Stenocereus griseus*, *Cereus repandus*, and *Pereskia guamacho* (Cactaceae). Other common species belong to the following genera: *Euphorbia*, *Croton*, *Panicum*, *Paspalum*, *Digitaria*, and *Opuntia* (Cumana 1999, González *et al* 2004).

### Ant sampling

Three sampling stations below 100 m of altitude were chosen in the westernmost 35 km of the peninsula, from Caimancito to Araya town (A, B, C; Fig 1). Each station corresponds to a 6-km lineal plot with five parallel transects of 130-m length, 10-m width, and 1.5-km separation between each other. All transects were located randomly in savannas or xerophilous shrublands in order to avoid flooded and unsuitable conditions for ground-dwelling ants (e.g., coastal plains and mangroves). Ants were sampled using ten pitfall traps along each transect with 13 m of spacing between each other. All traps were simultaneously active during *ca*. 72 h in June 2009. Traps were plastic containers with 90-cm diameter and 120 ml of capacity, filled with 70 ml of 70% ethanol, 11% monoethylene glycol and few drops of liquid soap. Therefore, the total sampling effort was 50 traps per sampling station, with 150 traps for the whole peninsula (ten traps × five transects × three sampling stations). Additionally, direct sampling was carried out on woody dominant vegetation (tree or shrub species) during trap installation (1000–1600 hours). At each transect ten individual plants with heights between 1.5 and 2.5 m were randomly selected and searched during 5 min. A total effort of 250 and 750 min of direct sampling was accomplished for each sampling station and the peninsula, respectively (5 min × 10 plants × 5 transects × 3 sampling stations). Ants were sorted and identified to species or morpho-species level using taxonomic keys for the Neotropical region (Palacio & Fernández 2003) and comparing them with museum specimens from the Museo del Instituto de Zoología Agrícola (MIZA), Universidad Central de Venezuela (UCV), Maracay—Venezuela. Voucher specimens were deposited in the MIZA.

### Richness estimation and data transformation

The EstimateS 8.2 (Colwell 2009) application was used to calculate second-order Chao (Chao2) and first-order Jackknife (JK1) estimators for each sampling station and the peninsula as a whole. These estimators take into account both, species with high frequency of capture and species with low frequency of occurrence considered as unique in incidence data terms (Colwell *et al* 2004, Colwell 2009). All estimations used incidence data with 100 randomizations of sample order.

Relative abundance in traps was standardized to reduce the marked variations among species records. First, adjusted abundance (AA) was calculated to evaluate ant abundance at sampling station level using the following formula: $\mathrm{AA}=(A)\times \left( {{O \left/ {100 } \right.}} \right)$, being *A* the total number of individuals of each species, and *O* the occurrence or percentage of traps in which each species is present (Lindsey & Skinner 2001). This method combines abundance with percentage of occurrence in a value and reduces the effect of traps being near nests or trunk trails (Lindsey & Skinner 2001, Pérez-Sánchez 2007, Pérez-Sánchez *et al* 2012). Second, we generated a data matrix calculating the natural logarithm of the abundance of species plus 1 [Ln (*A* + 1)] per trap in order to explore ant abundance variation along the aridity gradient, and perform further multivariate analysis.

### Gradient analysis

Since the aridity gradient in Araya parallels longitudinal change throughout the study area, we considered the east to west transition as a proxy of the gradient. Therefore, the geographic coordinates in meters of each trap and transect were used as spatial location data according to the Universal Transverse Mercator system (UTM, zone 20) (Fig 1). Linear and polynomial regression analyses were performed between the spatial location of each transect (independent variable) and ant diversity parameters (dependent variables) in order to establish the pattern of variation in the ant assemblage along the gradient. Ant richness was considered as the sum of species or genera records obtained by both sampling methods per transect, while relative abundance was data exclusively taken from traps considering the collecting methods as complementary rather than additive for abundance estimation (Bestelmeyer *et al* 2000).

Mantel correlograms were constructed for establishing the variation in species composition (β diversity) per spatial location of each trap. Dividing Mantel correlations into classes of discrete geographical distances, this analysis makes it possible to establish if geographically closest samples show higher similarity in their composition (Oden & Sokal 1986, Legendre & Legendre 1998). In our case, the main advantage of this analysis is to determine the geographic distance at which species composition (measured as dissimilarity) changes within ant assemblages. Bray–Curtis dissimilarity values for ant species were used as biotic matrix (dependent variable) and location data (*x*, *y*) as geographical matrix (independent variable). Spearman coefficient and Mantel statistic with permutation tests were used due to the nonparametric behavior of the data. Dissimilarity calculations, Mantel correlograms, and permutations tests were performed using vegdist and mantel.correlog commands included in the Vegan package of the R 2.13.2 statistical program (R Development Core Team 2011, Oksanen *et al* 2011).

Additionally, a two-indicator species analysis (TWINSPAN) was performed using an abundance matrix of species per traps. This method is a divisive and hierarchical classification that produces successive dichotomies in the formed groups from the weighted averages of species and samples (Legendre & Legendre 1998, McCune & Mefford 1999). This feature allows us to sort simultaneously both, species and groups of traps (sample units, SU) along the studied gradient. We conducted this analysis using the PC-ORD 4.0 statistical package (McCune & Mefford 1999).

## Results

We recorded about 40,000 ant specimens from 52 species, 23 genera, and 7 subfamilies [“Electronic supplementary material” (ESM) 1]. Overall, 49 species and 23 genera were recorded using pitfall traps, while 20 species and 11 genera were detected through direct sampling (Table 1, ESM 1). Only three species were recorded exclusively by manual technique (ESM 1). The total number of species recorded for the peninsula was close to the values predicted by both estimators (Table 1), indicating that about 95% of terrestrial ants and more than 77% of arboreal and diurnal species were collected in the peninsula. Ant genera and species richness decreased from sampling stations A to C, with the highest terrestrial and arboreal ant richness in the eastern peninsula (station A; Table 1). Although observed richness values are close to those predicted by the estimators of both sampling techniques, Chao2 estimation and confidence boundaries differ from JK1 predicted values, suggesting that at least ten species of arboreal ants eluded capture in station A (Table 1). Such differences between estimators are due to the high number unique and the absence of duplicates, which increase Chao2 in relation to JK1 (Colwell 2009). Additionally, genera richness showed a strong decrease from east to west in a similar way that species richness values (Table 1).

**Table 1:** Richness of ant species and genera along the aridity gradient.

Richness	Peninsula		Station A		Station B		Station C	
	Species	Genera	Species	Genera	Species	Genera	Species	Genera
Traps collect								
Observed	49	23	42	22	30	15	21	12
Chao2	52 ± 3	24 ± 2	45 ± 3	23 ± 1	33 ± 3	15 ± 1	26 ± 6	12
JK1	56 ± 3	26 ± 2	49 ± 4	25 ± 2	35 ± 2	17 ± 1	26 ± 2	13 ± 1
Direct sampling								
Observed	20	11	20	11	11	9	6	6
Chao2	26 ± 6	11	32 ± 10	13 ± 3	13 ± 4	9	7 ± 2	5
JK1	26 ± 3	11	30 ± 4	14 ± 2	13 ± 1	10 ± 1	8 ± 1	6 ± 1
Total	52	23	46	23	31	16	22	12

Number of species and genera observed are estimated (±SD) at the peninsula and sampling station level.

*Chao2* Chao second order results, *JK1* Jackknife first order results.

**Fig 2:**
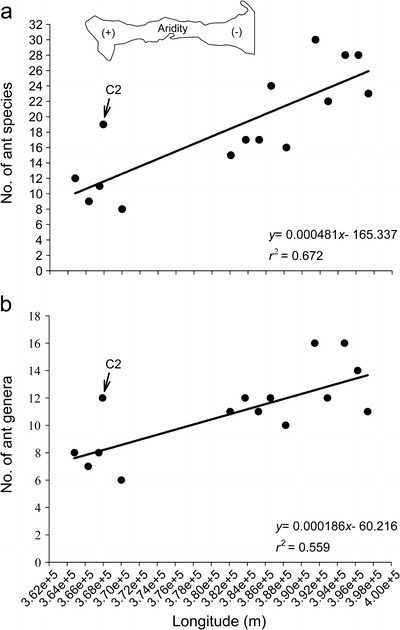
Relationship between ant species richness and longitudinal variation (aridity gradient) in Araya Peninsula, State of Sucre, Venezuela. Each *point* represents the total richness values of ant species (**a**) and genera (**b**). The *line* is a best fit regression model. The acronyms *C2* indicates the values recorded in the second transect of sampling station C.

**Fig 3:**
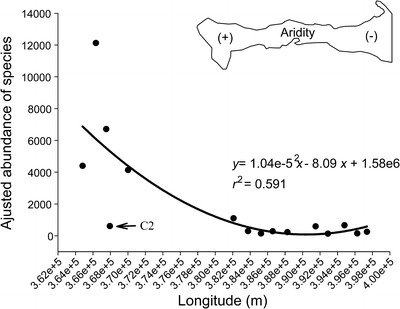
Relationship between the species adjusted abundance and longitudinal variation (aridity gradient) in Araya Peninsula, State of Sucre, Venezuela. Each *point* represents the adjusted abundance values of species in traps. The *line* corresponds to the regression model that best fit the data (quadratic polynomial regression). The acronym *C2* indicates the AA values recorded in the second transect of sampling station C.

Species and genera richness for each transect showed a significant linear decrease from east to west (*P* < 0.005; Fig 2), with specific richness, the parameter that best fits the linear model (*F* = 26.65, *df* = 15, *P* = 0.0002; Fig 2a). Although genera richness showed significant correlations with trap spatial arrangement, the association between both variables barely exceeds 55% (*P* < 0.005; Fig 2b). In terms of abundance, the AA values of species described a significant non-linear (quadratic polynomial) increase along the spatial transition of the gradient (*P* = 0.004); however, the association between ant abundance and gradient was not high (59%) (Fig 3). Note that species richness and relative abundance of the second transect in sampling station C (C2) differs from those recorded at the same site (Figs 2 and 3). Since these results probably increase data variation and skewed the studied pattern, we reassessed the regression analyses excluding C2 data. The results showed a similar but more robust trend, where ant richness decreases linearly along with increasing aridity (*r* ^2^ = 0.77 for species, *r* ^2^ = 0.63 for genera, *P* < 0.05), while relative abundance maintains a non-linear (quadratic polynomial) increase from east to west (*r* ^2^ = 0.75, *P* = 0.004).

**Fig 4:**
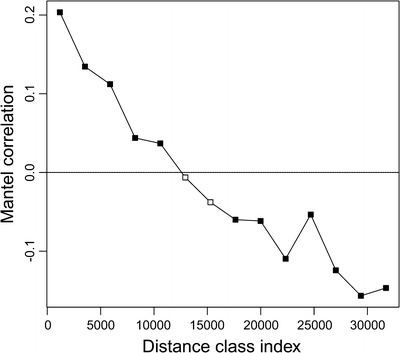
Mantel correlograms for the ant assemblages and longitudinal variation (aridity gradient) in Araya Peninsula, State of Sucre, Venezuela. Correlograms represent the spatial association pattern between ant composition and the geographical location of pitfall traps. The *filled boxes* represent the Mantel coefficient values that differ from the expected by chance (*p* < 0.05), the *empty boxes* represent the not significant Mantel statistic values (*p* > 0.05).

Significant spatial correlation values between β diversity and trap locations at five classes of discrete geographical distances were observed (*P* < 0.005), the association between both variables decreasing with increasing separation between samples (Fig 4). Mantel (Spearman) statistics were low (10–20%), but their significance and positive value indicated the mean similarity in the first classes of distance was greater than the mean similarity between the other classes of distance (Fig 4). Therefore, similarity in ant composition between traps was higher in those samples separated less than 11 km than for traps with wider separation (Fig 4).

**Fig 5:**
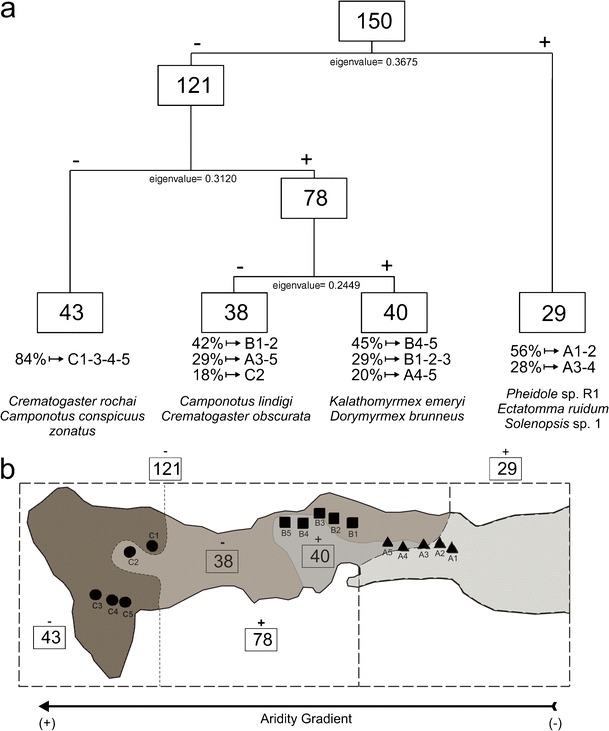
TWINSPAN classification analysis for the ant assemblage in Araya Peninsula, State of Sucre, Venezuela. **a** Classification of 49 species and 150 samples (pitfall traps), the *values in boxes* indicate the number of sample units (SU) per division, *percentages* indicate the contribution of samples from each transect, and the *names in italics* are the indicator species for each final SU, **b** Geographical representation of the TWINSPAN results within the peninsula, *dotted black lines* represent the division dichotomies and the *shades of grays* indicate the SU variation along the gradient.

TWINSPAN classification analysis generated three divisions, six groups of SU, and nine indicator species from the 49 species and 150 samples analyzed (pitfall traps) (Fig 5). In the first division, 29 samples were separated in the positive SU, mostly composed by samples from transects A1-2 and A3-4 (Fig 5a). Indicator species for this set were *Pheidole* sp. R1, *Ectatomma ruidum* (Roger), and *Solenopsis* sp. 1; while *Dorymyrmex brunneus* (Forel) and *Camponotus conspicuus zonatus* (Emery) characterized the 121 samples of the negative group. The second division occurred in the SU mentioned before (121 samples), where the positive branch showed 78 samples and the negative had 43 samples (Fig 5a). The indicators species for this dichotomy were *Solenopsis globularia* (Smith), *Acromyrmex rugosus* (Smith), and *Crematogaster obscurata* (Emery) for positive SU; whereas *Crematogaster rochai* (Forel) and *C*. *conspicuus zonatus* were indicators for the negative branch. No division was performed in the last negative group, mainly composed by traps from sampling station C (Fig 5a). The third split was performed in the last positive group (78 SU), resulting in two final groups of 38 SU (negative branch), and 40 SU (positive branch). In the negative branch, about 89% of the samples corresponded to data from transects B1-2, A3-5, and C2; with *Camponotus lindigi* (Mayr) and *C*. *obscurata* as indicator species (Fig 5a). In the positive group, the main contribution was from B4-5, B1-2-5, and A4-5 transects with *Kalathomyrmex emeryi* (Forel) and *D*. *brunneus* as indicator species (Fig 5a). The spatial distribution of the SU generated by TWINSPAN complements the Mantel correlogram results and showed changes in the composition and abundance of ants along the gradient (Fig 5b). Note that as in the regression analyses, data from C2 differ in respect to the other transects within the sampling station C (Fig 5b).

## Discussion

This work represents the first systematic survey of ants in the Araya Peninsula, and shows similar patterns to that observed in vegetation and herpetofauna richness in the region (Cumana 1999, González *et al* 2004). The variation in ant richness and relative abundance along the peninsula suggests the existence of an aridity gradient, at least in the westernmost 35 km. Considering the decrease in precipitation and net primary productivity as indicators of aridity, our results seem to match those reported for ant assemblages in arid locations of Africa and EEUU (Davidson 1977, Marsh 1986, Andersen 1997, Kaspari *et al* 2000, Sanders *et al* 2003), but contrast with those found in deserts and semi-deserts of Australia, Asia, and South America (Morton & Davidson 1988, Medel 1995, Pfeiffer *et al* 2003, Delsinne *et al* 2010). This ambiguity may be explained by Delsinne *et al* (2010) considerations on ant diversity and rainfall occurrence relationship in arid environments. These authors argue that the decrease of rainfall in arid environments does not exert negative effects on alpha diversity at regional and intercontinental scales (variations over of 400 km), while local studies in environments with low rainfall (>300 mm) show ant diversity decreases with increasing aridity (Davidson 1977, Marsh 1986, Sanders *et al* 2003). Therefore, our results agree with Delsinne *et al* (2010) due to local extension of the gradient (<100 km) and the erratic precipitation regime (>500 mm) in the Araya Peninsula.

Although rainfall variation is a common denominator in gradients of aridity, the gradient in Araya responds to a combination of atmospheric and topographic factors, rather than decline in rainfall per se (Ewel & Madriz 1976, Cumana 1999). In contrast to surrounding mesic locations (e.g., Paria Peninsula), the low relief of Araya offers less resistance to passing air masses (trade, sea, and land winds) which move and intensify along the same longitudinal direction of the peninsula (Ewel & Madriz 1976, Cumana 1999). These air masses absorb and transport humidity from lowlands at the northern border of the peninsula to the western tip and central-eastern elevations, contributing with the decrease in relative humidity and erratic behavior of rainfall (Ewel & Madriz 1976, Cumana 1999). Consequently, besides a longitudinal gradient of aridity, there is also a decrease of xeric conditions with increased elevations due to a rainfall shadow effect (Ewel & Madriz 1976, Cumana 1999). In this case, we would expect an increase in ant diversity along with elevation, where lower temperatures and higher precipitations may support higher levels of primary production and favorable habitat conditions for ants (Araújo & Fernandes 2003, Sanders *et al* 2003, Pérez *et al* unpublished data). Therefore, the complexity of vegetation and animal assemblages is expected to decrease proportionally with altitudinal and longitudinal aridity changes in Araya. Although elevation may be favorably conditioning habitat for ants, our survey did not contemplate altitudinal variation and all sampling stations were located below 150 m of altitude. Therefore, further studies would be needed to explore such altitudinal relationship.

All sampling levels (station, transect, trap, and vegetation) showed a decrease in ant richness along the aridity gradient for both, species and genera. Species richness was the best gradient descriptor as it decreased linearly with the gradient, indicating higher resource availability and favorable microhabitat conditions in the base than the western region of the peninsula. Spatial analyses indicate species turnover may be explained by spatial location (indirect measure of the aridity gradient) in the first classes of distances, which means that changes in ant composition can be detected every 11 km of distance. This is also supported by the turnover of indicator species and the overlap between the multivariate classification and spatial gradient. For example, *Pheidole* sp. R1 and *Solenopsis* sp. 1 are found in less arid habitats of eastern Araya and were absent in the rest of the region, while *A*. *rugosus* was representative of the middle gradient due to their high frequency in traps from intermediate sites, confirming its affinity to xeric regions with intermediate or disturbed vegetation (Pérez-Sánchez 2007). Finally, *D*. *brunneus*, *C*. *conspicuus zonatus*, and *C*. *rochai* were indicator species for the rest of the region due to their ubiquity throughout the peninsula.

Our results indicate an increased numerical dominance of *C*. *rochai* and *C*. *conspicuus zonatus* paralleling the longitudinal gradient. This high incidence and relative abundance in the most arid part of Araya can be based on their natural history and the absence of behavioral dominant ants such as *Solenopsis geminata* or *Pheidole fallax* in the west side of the peninsula (Andersen 1995, 1997, Reznikova 2003, Pérez-Sánchez 2007, Pérez-Sánchez *et al* 2012). *Crematogaster rochai* has polydomic nests, diurnal foraging activity, and moderately aggressive massive recruitment (Longino 2003), which may favor greater accessibility to resources as well as habitat colonization by expansion (clonal expansion sensu Stanton *et al* 2002). Therefore, we expect *C*. *rochai* to be a successful forager and colonizer in sites with reduced arboreal coverage. The presence of this species in traps suggests they explore other substrates due to limited habitat and resource availability (vegetation) in the harsh part of the gradient. The success of *C*. *conspicuus zonatus* could be explained by their nocturnal foraging and high propensity to relocate their nests (sensu Longino 2004, Pérez-Sánchez pers. obs.). Both behaviors have been related to avoidance of extreme temperatures and aggressive encounters with other species in xeric environments (Andersen 1995, Bestelmeyer 2000). Thus, the coexistence of both species in the arid tip of the region could be based on the temporal (day/night) and spatial (vegetation/soil) niche separation, mediated by interspecific competition and extreme arid conditions in the western Araya (Andersen 1995, Bestelmeyer 2000, Reznikova 2003).

All tests revealed “abnormal” behavior of the data in C2 and with similarities to ant assemblages from eastern more mesic environments (high species richness and evenness). We attribute this result to its location within an alluvial fan with dense and relatively wide vegetation (≈800 m^2^), which could favor colonization and establishment of a more complex myrmecofauna (Pérez-Sánchez 2007, Pérez-Sánchez *et al* 2012). Even though vegetation, soil properties, and topography were not considered in this paper, we propose that the combined action of these factors could explain specific changes in the ant fauna within the aridity gradient (sensu Mackay *et al* 1986, Bestelmeyer & Wiens 2001, Pérez-Sánchez 2007, Pérez-Sánchez *et al* 2012). The existence of two dominant floral communities, cactus scrubs and thorny shrublands, and the association of the latter with valleys and alluvial fans on the western tip of the peninsula support this argument (Cumana 1999, González *et al* 2004, Pérez-Sánchez *pers*. *obs*.).

In summary, our results suggest changes in ant composition every 11 km of distance with a clear decrease in species and genera richness, as well as the intensification of numerical dominance with the increasing longitudinal gradient (a proxy for the aridity gradient). Although our results cannot be explained entirely by the aridity gradient, we think microhabitat changes imposed by climate and topography exert a significant pressure on the ant fauna of the region. We consider that the evaluation of microhabitat heterogeneity (vegetation, soil, and temperature) and ant assemblage relationships will allow to accurately discern the factors that mold the ant fauna along the aridity gradient in the Araya Peninsula.

## Electronic supplementary material

Below is the link to the electronic supplementary material.

ESM 1 (DOC 271 kb)
