# Supplementary material for: Patterns of Ant (Hymenoptera: Formicidae) Richness and Relative Abundance along an Aridity Gradient in Western Venezuela
Source: Neotrop Entomol. 2012 Dec 21;42(2):128–36. doi: 10.1007/s13744-012-0096-y (PMC3601267; doi:10.1007/s13744-012-0096-y)
Supplement: Supplementary file 1 — (DOC 271 kb) [file 13744_2012_96_MOESM1_ESM.doc]

Appendix I. Ant richness and adjusted abundance per transect along the evaluated gradient in the Araya Peninsula, Venezuela. HC means hand collects.

| **Subfamily** | | **Sampling station and transects** | | | | | | | | | | | | | | |
| --- | --- | --- | --- | --- | --- | --- | --- | --- | --- | --- | --- | --- | --- | --- | --- | --- |
|  | **Species** | **A1** | **A2** | **A3** | **A4** | **A5** | **B1** | **B2** | **B3** | **B4** | **B5** | **C1** | **C2** | **C3** | **C4** | **C5** |
| Dolichoderinae | |  |  |  |  |  |  |  |  |  |  |  |  |  |  |  |
| *Azteca* sp. 1A | |  | HC |  |  |  |  |  | HC |  |  |  |  |  |  |  |
| *Dorymyrmex brunneus* (Forel) | | 1.6 | HC | 26.4 | 5.1 | 41.4 | 77.4 | 32.4 | 76.8 | 78.4 | 417 | 107.6 | 0.2 | 160.8 | 9 | 1228.5 |
| Formicinae | |  |  |  |  |  |  |  |  |  |  |  |  |  |  |  |
| *Brachymyrmex* sp. 1 | |  |  | 2.7 |  | 0.4 | 0.1 | 7 | 0.6 | 3 | 6.4 |  | 0.2 | 0.8 | 1 | 20.4 |
| *Brachymyrmex* sp. 2 | | 35.7 |  |  |  |  |  | 10.2 |  | 1.6 |  |  |  |  |  |  |
| *Camponotus atriceps* (Smith) | | 0.1 |  |  | 0.1 | 4.4 |  | 0.2 | 0.4 |  |  |  | 0.1 |  |  |  |
| *Camponotus conspicuus zonatus* (Emery) | | 8.5 |  |  |  | 14 | 5.4 | 64 | 8.4 | 21.7 | 284 | 841.5 | 219 | 4202.1 | 4468 | 946 |
| *Camponotus lindigi* (Mayr) | | 14.4 | 2.7 | 13.2 | 32.2 | 53.9 | 68.4 | 42 | 16 | 3.3 |  | 10.2 | 59.4 | 7.2 | 1.8 | 784 |
| *Camponotus c.f. sanctaefidei* (Dalla Torre) | | 0.1 | 0.6 | 24.5 |  |  |  |  |  |  |  |  |  |  |  | 0.1 |
| *Camponotus* sp. 2 | |  |  | 0.1 |  | 0.4 |  |  |  |  |  |  |  |  |  |  |
| *Camponotus* sp. 3 | |  |  |  | 0.6 | 0.6 |  |  |  |  |  |  |  |  |  |  |
| *Camponotus* sp. 4 | |  | HC |  |  |  |  |  |  |  |  |  |  |  |  |  |
| *Nylanderia fulva* (Mayr) | |  |  |  |  |  |  | 0.5 |  |  |  |  |  |  |  |  |
| *Paratrechina longicornis* (Latreille) | | 0.2 | 14.5 | 2.1 | 6.5 | 6 | 0.2 |  |  | 96 | 1.5 | HC | 3.5 |  | 0.4 | 60.8 |
| Pseudomyrmicinae | |  |  |  |  |  |  |  |  |  |  |  |  |  |  |  |
| *Pseudomyrmex cuaracaensis* (Forel) | |  | HC | 0.4 |  |  |  | HC |  |  | 0.2 |  |  | HC |  |  |
| *Pseudomyrmex termitarius* (Smith) | |  |  |  |  |  |  | 7.2 |  |  |  |  | 2.4 |  |  |  |
| *Pseudomyrmex* *simples* (Smith) | |  | HC |  |  |  |  | 0.1 | 0.1 |  |  |  |  |  |  |  |
| *Pseudomyrmex* sp. 3L | |  |  |  |  |  |  | 0.1 | 0.1 |  | 0.1 |  |  | 0.1 |  |  |
| Ecitoninae | |  |  |  |  |  |  |  |  |  |  |  |  |  |  |  |
| *Labidus coecus* (Latreille) | |  | 5 | 0.2 |  |  |  |  |  |  |  |  |  |  |  |  |
| *Neivamyrmex humilis* (Borgmeier) | |  | 4.2 |  |  | 0.9 |  |  |  |  |  |  |  |  |  |  |
| Ectatomminae | |  |  |  |  |  |  |  |  |  |  |  |  |  |  |  |
| *Ectatomma ruidum* (Roger) | | 5.4 | 11.4 | 32.8 | 10.2 | 1.16 |  | 0.6 | 10.5 |  |  |  | 14 | 0.1 |  |  |
| Ponerinae | |  |  |  |  |  |  |  |  |  |  |  |  |  |  |  |
| *Anochetus emarginatus* (Fabricius) | |  |  | 0.1 |  |  |  |  |  |  |  |  | 0.1 |  |  |  |
| *Anochetus* sp. 1 | |  |  |  |  |  |  |  |  | 0.1 |  |  |  |  |  |  |
| *Leptogenys* sp. 1 *pubiceps* complex | | 0.1 |  | 0.1 |  |  |  |  |  |  |  |  |  |  |  |  |
| *Odontomachus bauri* (Emery) | | 4 | 1.2 |  |  | 0.1 |  |  |  |  |  |  |  |  |  |  |
| *Plathytyrea* sp. 1 | |  |  |  |  | 0.1 |  |  |  |  |  |  |  |  |  |  |

*Continuation*

| Myrmicinae |  |  |  |  |  |  |  |  |  |  |  |  |  |  |  |
| --- | --- | --- | --- | --- | --- | --- | --- | --- | --- | --- | --- | --- | --- | --- | --- |
| *Acromyrmex rugosus* (Smith) |  |  |  | 0.6 | 36.8 | 22.4 | 17.2 |  | HC | 18.6 |  |  |  |  |  |
| *Cardiocondyla emeryi* (Emery) |  |  |  | 0.4 |  |  |  |  |  |  |  |  |  |  |  |
| *Cardiocondyla minutior* (Emery) |  |  |  | 0.3 | 1.4 |  |  |  |  |  |  |  |  |  |  |
| *Cephalotes decolor* (De Andrade) | 0.1 |  | 0.1 |  |  | 0.3 |  | 0.6 | 0.1 | 0.4 |  | 0.4 |  |  |  |
| *Cephalotes maculatus* (Smith) |  | HC |  |  |  |  |  |  |  |  |  |  |  |  |  |
| *Cephalotes* *pusillus* (Klug) | 4 | 0.1 | 18.2 | 2.4 | 28.8 | 0.1 | HC | 0.9 | 3.1 | 1.2 |  |  |  |  |  |
| *Crematogaster obscurata* (Emery) | 0.1 | HC | 0.8 |  | 3.5 | 0.1 | 11.4 | 0.2 | 0.6 | 0.1 |  | 8.4 |  |  |  |
| *Crematogaster* *rochai* (Forel) | HC | HC | 132 | 0.1 |  | 15.2 | 0.1 |  |  |  | 3159 | 0.3 |  | 7622.1 | 3564 |
| *Cyphomyrmex* sp. 1 *rimosus* complex |  | 0.1 | 0.1 |  |  |  |  |  |  |  |  |  |  |  |  |
| *Cyphomyrmex* sp. 2 |  |  |  |  |  |  |  |  |  |  |  |  |  |  |  |
| *Kalathomyrmex emeryi* (Forel) |  |  | 4.4 | 1 | 1 | 0.6 | 0.8 | 1.5 | 1.8 | 110 |  | 0.4 | 21.7 |  | 0.9 |
| *Pheidole fallax* |  |  | 1.2 | 0.3 | 24 |  |  |  |  |  |  |  |  |  |  |
| *Pheidole* sp. F1 *fallax* complex |  | 23.7 |  | HC | 0.1 |  |  |  |  |  |  | 238 |  |  |  |
| *Pheidole* sp. R1 *radoszkowskii* complex | 121.1 | 49.5 | 263.2 | 46 | 239.6 | 2.2 | 0.8 | 1 | 34.2 |  |  |  |  |  |  |
| *Pheidole* sp. R2 *radoszkowskii* complex | 14 | 0.8 | 7.2 | 3.5 | 51.1 | 7 | 6.6 | 4.5 | 33.6 | 13 | 16.1 | 12 | 3.2 | 21 | 5.4 |
| *Pheidole* sp. R3 *radoszkowskii* complex | 8.4 | 3 | 1.8 | 15.2 | 39.9 | 4 | 31.8 | 0.6 | 11.4 | 156 | 0.1 | 13.5 | 3.3 | 1.2 | 97.2 |
| *Pheidole* sp. R5 *radoszkowskii* complex | 0.1 | 4.8 | 3.2 | 2 | 5.4 |  | 0.4 |  |  |  |  | 0.1 |  |  |  |
| *Pheidole* sp. R6 *radoszkowskii* complex |  | 0.2 |  |  |  |  | 1.2 |  |  |  |  |  |  |  |  |
| *Pheidole transversostriata* (Mayr) | 0.4 | 4 |  |  | 0.5 |  |  |  |  |  |  |  |  |  |  |
| *Solenopsis geminata* (Fabricius) | 12.4 | 10 | 80.5 | 0.2 | 4.5 | 16 | 51.1 |  |  |  |  | 30 | 0.4 |  |  |
| *Solenopsis globularia* (Smith) | 10.8 | 2.4 | 44 | 5 | 32.8 | 9 |  | 21.6 | 3.6 | 91 | 2 | 7 | 0.6 | 0.4 | 4 |
| *Solenopsis* sp. 1 | 6.9 | 10.8 | 2 | HC | 0.4 |  |  |  |  |  |  |  |  |  |  |
| *Solenopsis* sp. 2 | 1.2 | 0.6 | 0.1 |  |  |  | 0.1 |  |  |  |  |  |  |  |  |
| *Solenopsis* sp. 3 |  |  |  |  |  |  |  |  |  | 1.2 |  |  |  |  |  |
| *Tetramorium simillimum* (Smith) |  |  |  | 0.6 | 0.1 |  |  |  |  |  |  |  |  |  |  |
| *Trachymyrmex* sp. |  |  | 0.3 |  |  |  |  |  | 0.3 |  |  |  |  |  |  |
| Morphospecies 1 |  |  | 0.47 |  |  |  |  |  |  |  |  |  |  |  |  |
| **Number of species** | **23** | **28** | **27** | **22** | **30** | **16** | **24** | **17** | **17** | **15** | **8** | **19** | **12** | **9** | **11** |
| **Number of genera** | **10** | **14** | **16** | **12** | **16** | **10** | **12** | **11** | **12** | **10** | **6** | **11** | **8** | **7** | **8** |
| **Adjusted abundance of species** | **249.6** | **149.6** | **661.7** | **132.3** | **593.3** | **228.4** | **285.8** | **143.8** | **292.8** | **1100.7** | **4136.5** | **609** | **4400.3** | **12125** | **6711.3** |
